# Supplementary material for: The COMPASS subunit Spp1 protects nascent DNA at the Tus/Ter replication fork barrier by limiting DNA availability to nucleases
Source: Nat Commun. 2023 Sep 5;14:5430. doi: 10.1038/s41467-023-41100-4 (PMC10480214; doi:10.1038/s41467-023-41100-4)
Supplement: Supplementary file 1 — Supplementary Information [file 41467_2023_41100_MOESM1_ESM.pdf]

Supplemental information

The COMPASS subunit Spp1 protects nascent DNA at the Tus/*Ter* replication fork barrier by limiting DNA availability to nucleases

Supplemental Figures

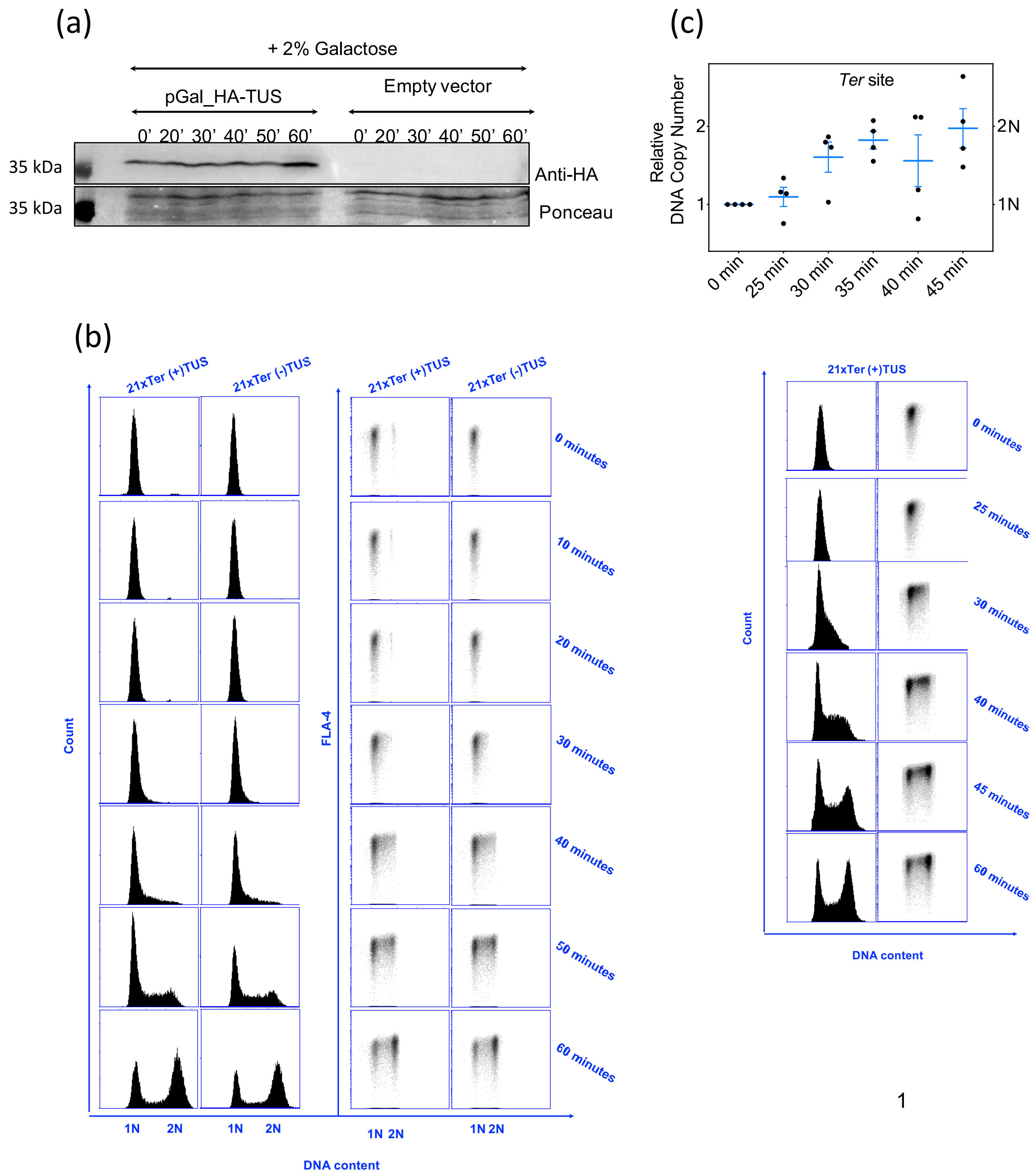

Supplemental Figure 1. Spp1 subunit is recruited to stalled replication fork in early replication

(**a**) Representative Western blot of Tus expression in the ChIP samples shown in Fig. 1. Samples were migrated on 12% SDS-PAGE gel and HA-Tus was revealed using an antibody against the HA tag. The western blot was repeated 3 times with reproducible results.

(**b**) Cell cycle progression profiles after release from alpha factor of strains expressing Tus (+TUS) or with an empty vector (-TUS). DNA content is visualized using Sytox Green. Early S phase occurs between 30 and 40 min.

(**c**) Top, Scatter dot blot shows the DNA copy number using qPCR with pair of primers covering the 9th and 10th *Ter* repeat. Data are normalized to a non-replicated region. Data are represented as mean value +/- SEM corresponding to n=4 independent experiments.

Source data are provided as a Source Data file.

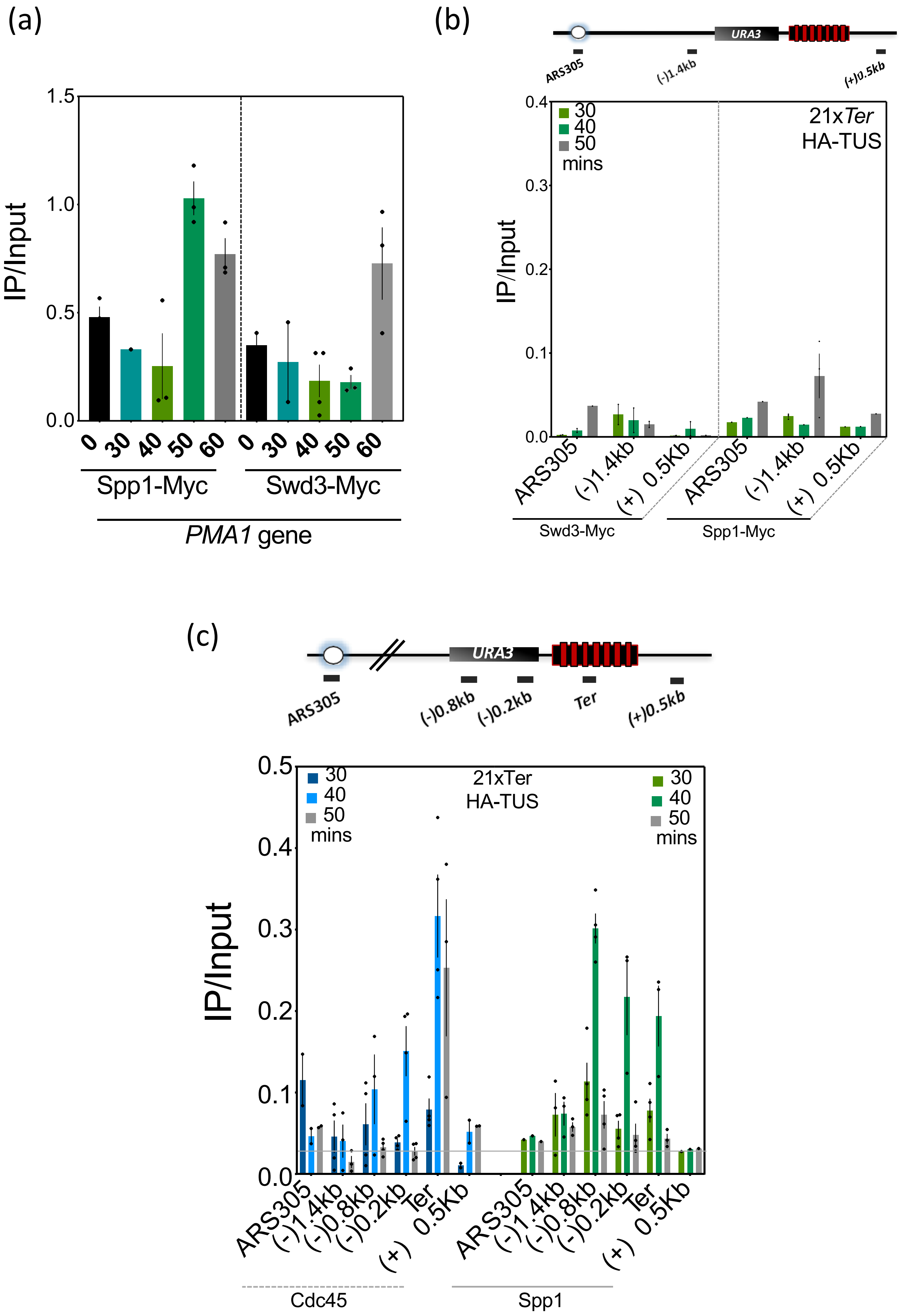
(a)
IP/Input
1.5
1.0
0.5
0.0
0 30 40 50 60 0 30 40 50 60
Spp1-Myc
Swd3-Myc
PMA1 gene
(b)
URA3
ARS305
(-)1.4kb
(+)0.5kb
0.4
0.3
0.2
0.1
0.0
30
40
50
mins
21xTer
HA-TUS
IP/Input
ARS305
(-)1.4kb
(+) 0.5Kb
Swd3-Myc
Spp1-Myc
(c)
URA3
ARS305
(-)0.8kb
(-)0.2kb
Ter
(+)0.5kb
0.5
0.4
0.3
0.2
0.1
0.0
30
40
50
mins
21xTer
HA-TUS
IP/Input
ARS305
(-)1.4kb
(-)0.8kb
(-)0.2kb
Ter
(+) 0.5Kb
Cdc45
Spp1

Supplemental Figure 2. Spp1 recruitment to stalled fork is independent of Set1C

(**a**) ChIP-qPCR of Spp1-Myc and Swd3-Myc at the highly transcribed gene *PMA1*. Data are represented as mean value +/- SEM corresponding to n=3 independent experiments.

(**b**) Top, schematic representation of the pair of primers used for ChIP-qPCR. Bottom, ChIP-qPCR profiles from the same samples described in Fig. 2b but at ARS305, (-) 1.4Kb, and (+) 0.5Kb relative to *Ter* site. The scale is the same as shown in Fig. 2b for the comparison. Data are represented as mean value +/- SEM corresponding to n=3 independent experiments.

(**c**) ChIP-qPCR of Cdc45-V5 and Spp1-Myc from the same chromatin at the different indicated regions and times in S phase. Data represent three independent experiments. Data are represented as mean value +/- SEM and correspond n=4 independent experiments.

Source data are provided as a Source Data file.

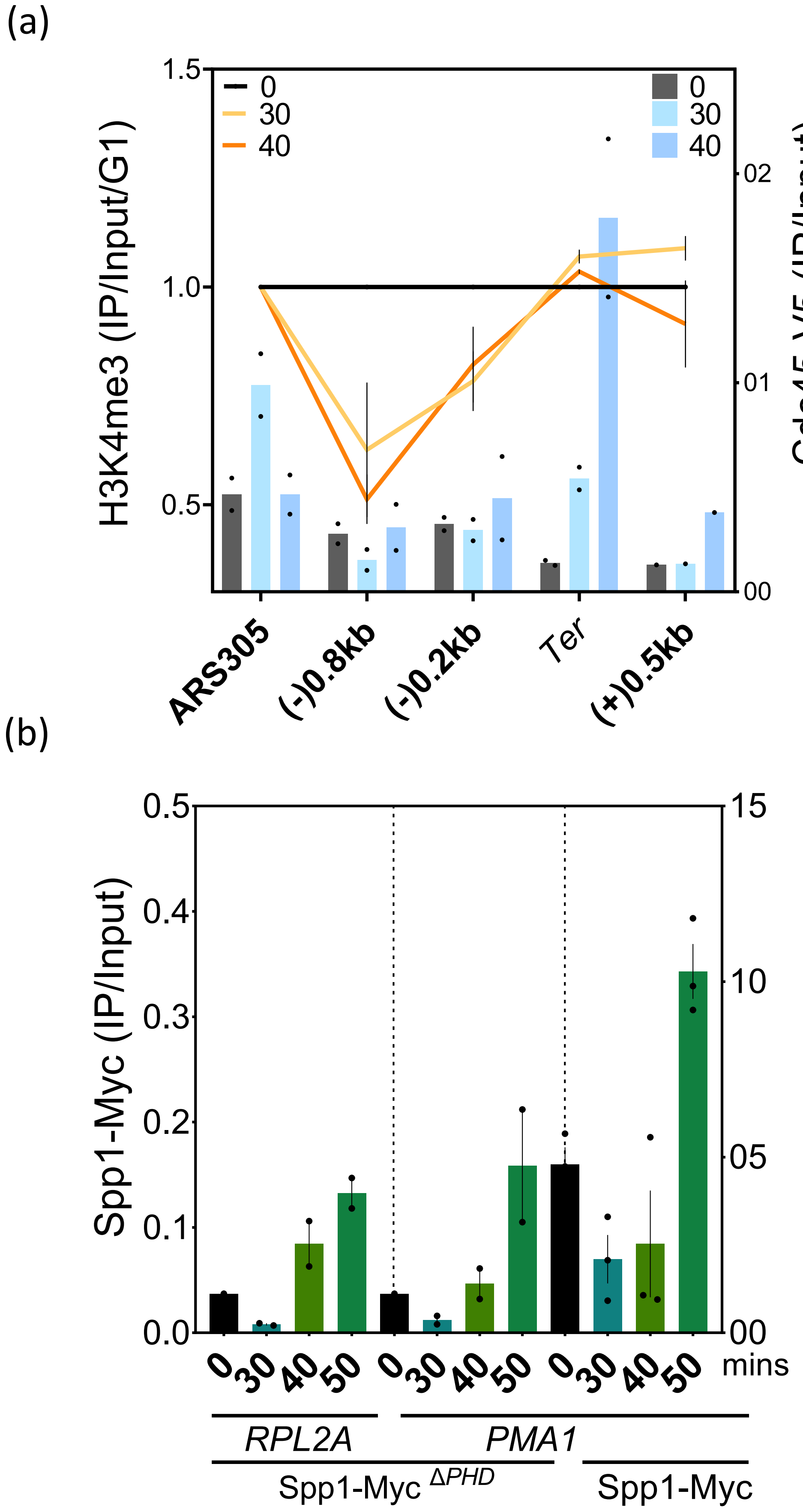

(a)
1.5
1.0
0.5
H3K4me3 (IP/Input/G1)
0
30
40
0
30
40
02
01
00
Cdc45-V5 (IP/Input)
ARS305
(-)0.8kb
(-)0.2kb
Ter
(+)0.5kb
(b)
0.5
0.4
0.3
0.2
0.1
0.0
Spp1-Myc (IP/Input)
15
10
05
00
0
30
40
50
0
30
40
50
0
30
40
50
mins
RPL2A
PMA1
Spp1-Myc ΔPHD
Spp1-Myc

Supplemental Figure 3. Spp1 and H3K4me3 distribution surrounding Tus/*Ter* barrier is dependent on its PHD finger domain

(**a**) ChIP profiles at the same regions of those shown in Fig. 3a. H3K4me3 (IP/input) is normalized to its amount in G1 to obtain clearer dilution of H3K4me3 mark during replication. Data are represented as mean value +/- SEM of n=2 independent biological replicates of H3K4me3 colored line graph and of Cdc45 in a blue-colored bar graph.

(**b**) ChIP-qPCR of Spp1-Myc and $Spp1^{\Delta PHD}$-Myc at the indicated genes. Data are presented as mean values of +/- SEM of n=3 independent experiments in WT (Spp1-Myc) and n=2 independent experiments in $spp1^{\Delta PHD}$.

Source data are provided as a Source Data file.

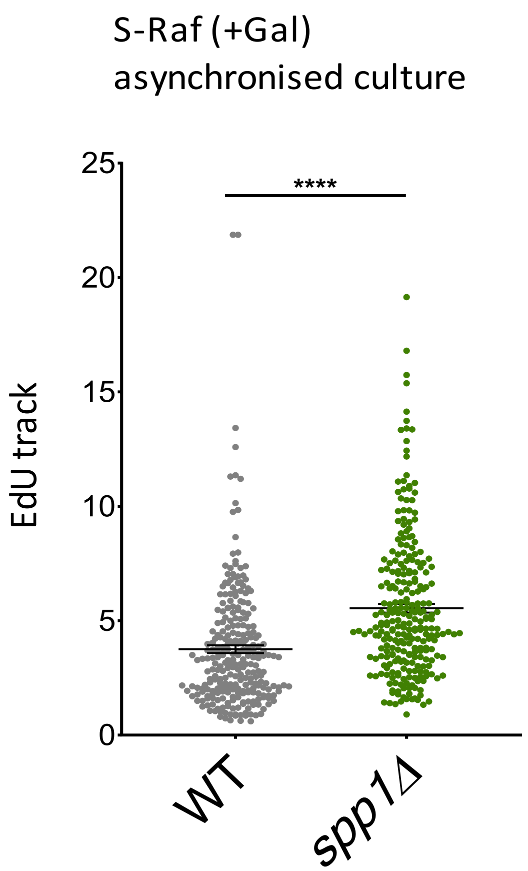

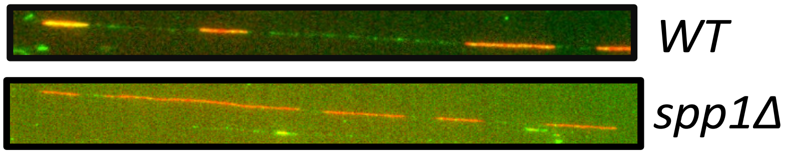

Supplemental Figure 4. Replication fork progression observed by DNA combing assay.

Top, Scatter dot plot shows the EdU track length measured in WT, and *spp1*Δ asynchronized cultures grown in S-Raffinose (+GAL) with 20 min pulse of EdU before sample collection. Statistical analysis of mean value +/- SEM was performed using a two-tailed Mann-Whitney test from n= 250 fibers examined over 3 independent experiments in each strain background, n.s., not significant. **p < 0.01, ****p < 0.0001. The red colour represents EdU incorporated DNA while green is unreplicated DNA.

Source data are provided as a Source Data file.

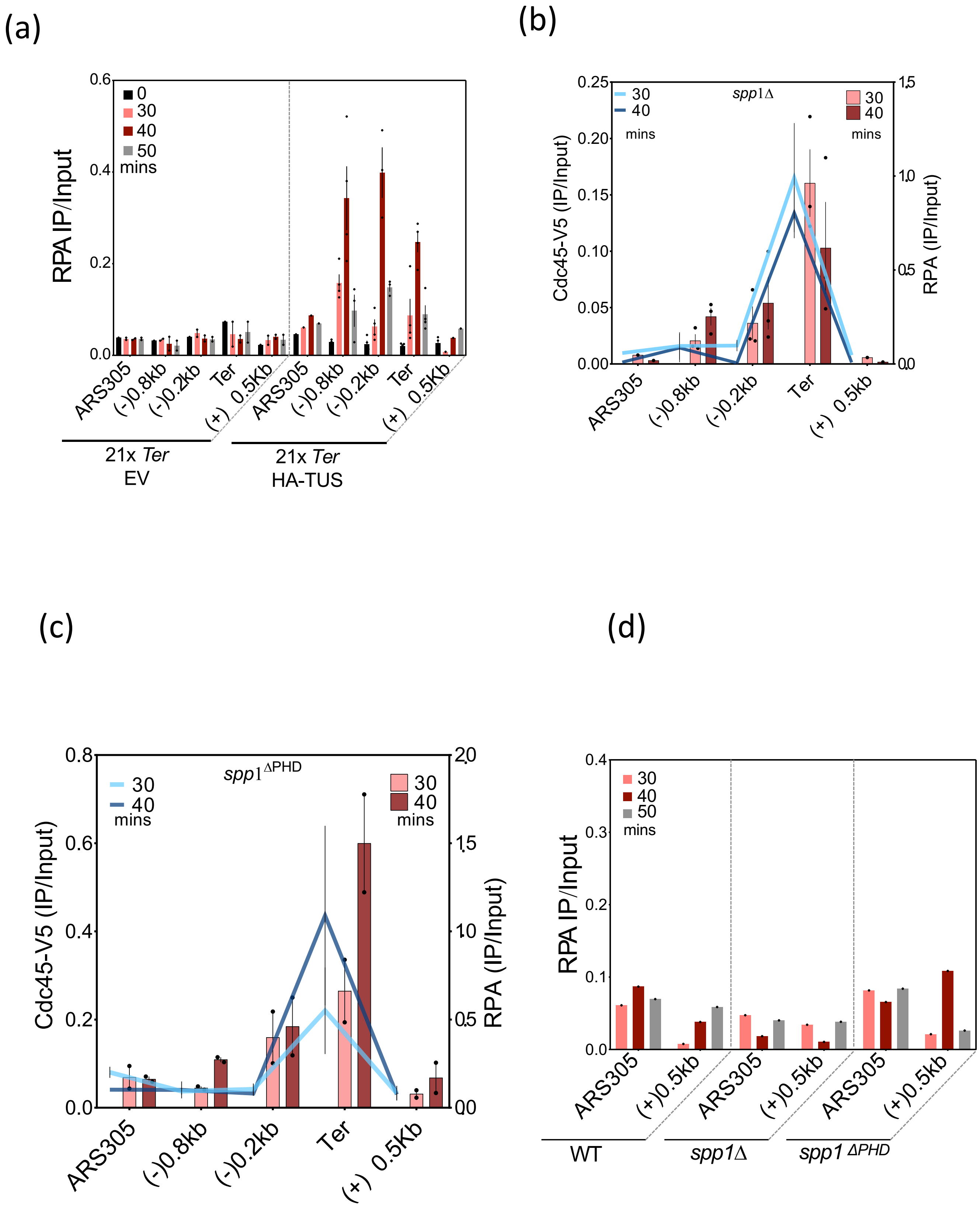

(a)
(b)
(c)
(d)
RPA IP/Input
0 30 40 50 mins
ARS305 (-)0.8kb (-)0.2kb Ter (+) 0.5Kb
21x Ter EV
21x Ter HA-TUS
Cdc45-V5 (IP/Input)
RPA (IP/Input)
spp1Δ
30 40 mins
spp1ΔPHD
30 40 50 mins
ARS305 (+)0.5kb
WT
spp1Δ
spp1 ΔPHD

Supplemental Figure 5. RPA occupancy at Tus/*Ter* dependent-stalled fork in the absence of Spp1 or its PHD finger domain

(**a**) ChIP-qPCR of RPA in strains expressing Tus protein (or not, EV) at the indicated regions. ChIP-qPCR samples are the same as those of Fig. 5a. Data are represented as mean value +/- SEM and correspond to n=4 biologically independent experiments for 21x*Ter* HA-TUS and n=2 biologically independent experiments for 21x*Ter* EV

(**b**) and (**c**) Overlapping ChIP-qPCR profiles of RPA and Cdc45 at all regions surrounding Tus/*Ter* barrier system in *spp1*Δ and $spp1^{\Delta PHD}$ strains, respectively. Both experiments were done in the same experimental conditions as previously described. The red bar graphs represent RPA level while the blue lines represent Cdc45-V5 levels. Data are represented as mean value +/- SEM and correspond to n=3 of biologically independent experiments for *spp1*Δ and n= 2 for biologically independent experiments $spp1^{\Delta PHD}$.

(**d**) RPA ChIP-qPCR profiles between WT, *spp1*Δ and $spp1^{\Delta PHD}$ at regions surrounding the Tus/Ter barrier. RPA levels were quantified with the same chromatin samples of those used for Cdc45.

Source data are provided as a Source Data file.

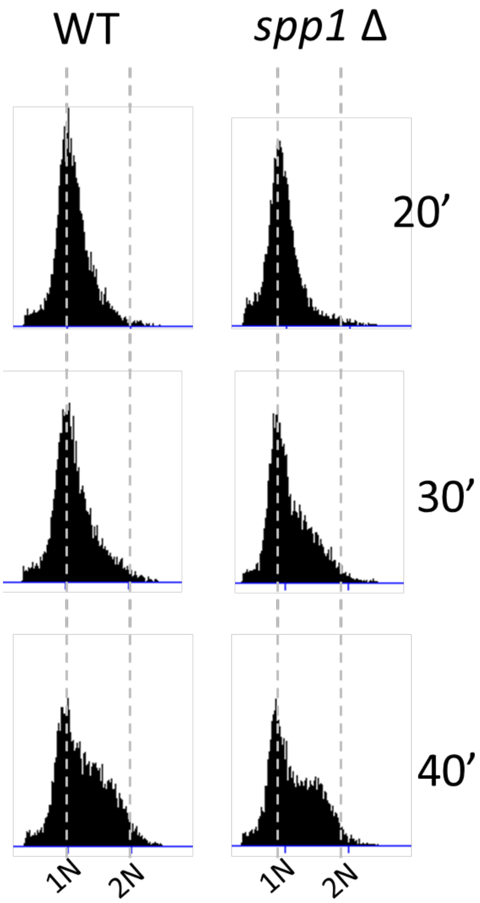

Supplemental Figure 6.

FACS profile in WT and *spp1*Δ corresponding to nascent chromatin accessibility assay experiment.

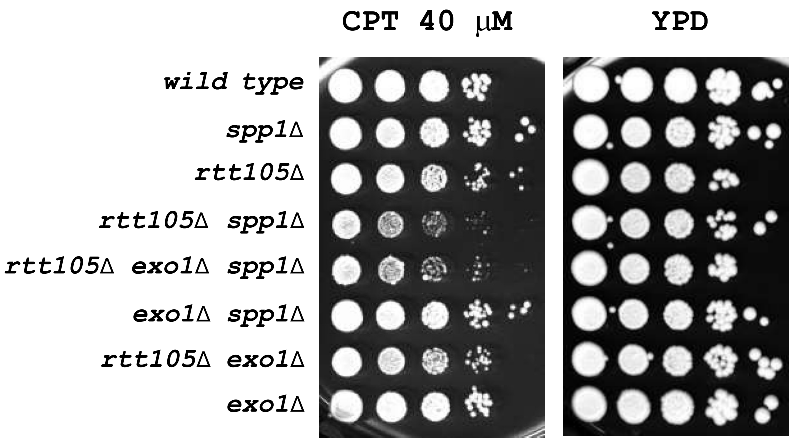

Supplemental Figure 7.

Genetic interactions between *rtt105*Δ and *spp1*Δ. Tenfold serial dilutions of exponentially growing cells with the indicated mutations were spotted onto YPD plates with or without CPT and incubated at 30° C for 3 days.

Supplemental Table 1: Yeast strains used in this study

Name	Genotype	Source
WT	MATa his-11,15 leu2-3,112, trp1-1, ura3-1, can1-100, ade2-1	VG collection
Tus/*Ter*	MATa his-11,15 leu2-3,112 trp1-1 ura3-1 can1-100 ade2-1 RAD5^{+} Cdc45-V5::TRP1 URA3::21xTer pG413-HA-TUS::HIS3	This study
EV/*Ter*	MATa his-11,15 leu2-3,112 trp1-1 ura3-1 can1-100 ade2-1 RAD5^{+} Cdc45-V5::TRP1 URA3::21xTer, pG413::HIS3	This study
Spp1-Myc Tus/*Ter*	MATa his-11,15 leu2-3,112 trp1-1 ura3-1 can1-100 ade2-1 RAD5^{+} Cdc45-V5::TRP1 URA3::21xTer Spp1-Myc::kanMX6 pG413-HA-TUS::HIS3	This study
Spp1-Myc EV/*Ter*	MATa his-11,15 leu2-3,112 trp1-1, ura3-1, can1-100 ade2-1 RAD5^{+} Cdc45-V5::TRP1 URA3::21xTer Spp1-Myc::kanMX6 pG413::HIS3	This study
Swd3-Myc Tus/*Ter*	MATa his-11,15 leu2-3,112 trp1-1 ura3-1 can1-100 ade2-1 RAD5^{+} Cdc45-V5::TRP1 URA3::21xTer Swd3-Myc::hphMX6 pG413-HA-TUS::HIS3	This study
spp1Δ	Mata his-11,15 leu2-3,112 trp1-1 ura3-1 can1-100 ade2-1 RAD5^{+} spp1Δ:: hphMX6	This study
spp1Δ Tus/Ter	MATa his-11,15 leu2-3,112 trp1-1 ura3-1 can1-100 ade2-1 RAD5^{+} Cdc45-V5::TRP1 URA3::21xTer Swd3-Myc::hphMX6 pG413-HA-TUS::HIS3 spp1Δ::hphMX6	This study
spp1^{ΔPHD}	MATa his-11,15 leu2-3,112 trp1-1 ura3-1 can1-100 ade2-1 Spp1^{ΔPHD}-Myc::kanMX6	VG collection
spp1^{ΔPHD} Tus/*Ter*	MATa his-11,15 leu2-3,112 trp1-1 ura3-1 can1-100 ade2-1 Spp1^{ΔPHD}-Myc::kanMX6 RAD5^{+} Cdc45-V5::TRP1 URA3::21xTer pG413-HA-TUS::HIS3	This study
PP2266 (EdU-Inc)	MATa Myc::kanMX6 URA3::GPF-TK(5x) AUR1c::ADH-hENT1 RAD5^{+}	Phillipe Pasero Lab
spp1Δ (EdU-Inc)	MATa his-11,15 leu2-3,112 trp1-1 ura3-1 can1-100 ade2-1 URA3::GPF-TK(5x) AUR1c::ADH-hENT1 RAD5^{+} spp1Δ::hphMX6	This study
spp1^{ΔPHD} (EdU-Inc)	MATa his-11,15 leu2-3,112 trp1-1 ura3-1 can1-100 ade2-1 URA3::GPF-TK(5x) AUR1c::ADH-hENT1 RAD5^{+} Spp1^{ΔPHD}-Myc::kanMX6	This study
set1Δ (EdU-Inc)	MATa his-11,15 leu2-3,112 trp1-1 ura3-1 can1-100 ade2-1 URA3::GPF-TK(5x) AUR1c::ADH-hENT1 RAD5^{+} set1::NATMX6	This study
YVC392 rtt105Δ	MATa his-11,15 leu2-3,112 trp1-1 ura3-1 can1-100 RAD5^{+} rtt105Δ::kanMX6	YC collection (VG lab)
YVC399 (rfa1-CFP)	MATa his-11,15 leu2-3,112 trp1-1 ura3-1 can1-100 ade2-1 rfa1-CFP RAD5^{+}	YC collection (VG lab)
spp1Δ rfa1-CFP	MATa his-11,15 leu2-3,112 trp1-1 ura3-1 can1-100 ade2-1 rfa1-CFP RAD5^{+} spp1Δ::hphMX6	This study

spp1$^{\Delta PHD}$ rfa1-CFP	MATa his-11,15 leu2-3,112 trp1-1 ura3-1 can1-100 ade2-1 rfa1-CFP Spp1$^{\Delta PHD}$-Myc::kanMX6 RAD5^{+}	This study
rtt105Δ spp1Δ	his-11,15 leu2-3,112 trp1-1 ura3-1 can1-100 rtt105Δ::kanMX6 spp1Δ::hphMX6	This study
YVC492 pCLB2-Rfa1	MATa his-11,15 leu2-3,112 trp1-1 ura3-1 can1-100 pCLB2-CLB2(1-180, L26A)-RFA1::natMX6	Richard Kolodner
pCLB6-Rfa1	MATa his-11,15 leu2-3,112 trp1-1 ura3-1 can1-100 pCLB6-CLB6(1-195)-RFA1::natMX6	Richard Kolodner
pCLB2-Rfa1*spp1Δ*	MATa his-11,15 leu2-3,112 trp1-1 ura3-1 can1-100 pCLB2-CLB2(1-180, L26A)-RFA1::natMX6 spp1Δ::hphMX6	This study
pCLB6-Rfa1*spp1Δ*	MATa his-11,15 leu2-3,112 trp1-1 ura3-1 can1-100 pCLB6-CLB6-RFA1 spp1Δ::hphMX6	This study
rfa1-D228Y	MATa his-11,15 leu2-3,112 trp1-1 ura3-1 can1-100 rfa1-d228y	YC collection (VG lab)
rfa1-D228Y spp1Δ	his-11,15 leu2-3,112 trp1-1 ura3-1 can1-100 rfa1-d228y spp1Δ::hphMX6	This study
rfa1-T11	MATalpha his-11,15 leu2-3,112 trp1-1 ura3-1 can1-100 rfa1-t11	Susan Gasser Lab
rfa1-T11 spp1Δ	his-11,15 leu2-3,112 trp1-1 ura3-1 can1-100 rfa1-t11 spp1Δ::hphMX6	This study
Rad52-YFP	his-11,15 leu2-3,112 trp1-1 ura3-1 can1-100 rad52-YFP	YC collection (VG lab)
Rad52-YFP *spp1Δ*	his-11,15 leu2-3,112 trp1-1 ura3-1 can1-100 rad52-YFP spp1Δ::hphMX6	This study

Supplemental Table 2: Oligonucleotides used in this study

**Name**	**sequence**
PMA1 5- OFS705	TCAGGTCATCAGCCAACTCAAG
PMA1 5- OFS706	CGTCGACACCGTGATTAGATTG
ARS_305_F	GGCCAGTTTGAATGCTCAACTC
ARS_305_R	GTTTTTAGCCCCCGTGTAAGTTAC
1000_F	GTCAAAGCCACAAAGAGGGAA
1000_R	GCAATTCCAGGGTTCAAGAGA
2000_F	TGTTCTAGCTACAGTCACTGC
2000_R	CTGCGTAGCATTTGGAGTAGT
3000_F	AACGCAAGAAGGTGAAGACTC
3000-R	ACATAGCAACAAGGCATGTACC

4000_F	GGATGGAGATCAACTGCGAAT
4000_R	CCATACACGCCAGTTATCCC
ARS_607_F	CGTGCGGCAGTATAAGTTCA
ARS_607_R	GCAGGATCGACCTGACTCTT
(-)1.4kb *Ter_R*	CCAATTCGCCCTATAGTGAGTCG
(-)1.4kb *Ter_F*	CATTCGGGTATCCCAGCTTTG
Ter 9	ACGTAACGGTAAGAGGCTCA
Ter 10	CGGACTCCTTATGCAGACAG
(-)800_F	GGATGTTCGTACCACCAAGG
(-)800_R	CTTAACTGTGCCCTCCATGG
(-)200_F	GTGGTCTCTACAGGATCTGAC
(-)200_R	CTCAAATATGCTTCCCAGCC
NegV_F	GCACTTAATTGGCGTAAGCTG
NegV_R	TCGCAGGAGCATATTTCGTA
TER_DONWSTREAM F	GCCTTTTCCCAAAGGGAAGG
TER_DONWSTREAM R	ATCTTCCCTAAAGGGTATCTTACG

Supplemental Table 3: Reagents used in this study

REAGENT	SOURCE	IDENTIFIER
Antibodies		
Anti-PK (anti-V5 tag)	Life Technologies	Cat#R960-25
Anti-HA	Santa Cruz	Cat#SC-7392
Anti-H3	Abcam	Cat#Ab1791
Anti-H3K4me3	EpiGentek	Cat# A-4033-100
Anti-RPA	Agrisera	Cat#AS07214
Anti-Myc (9E10)	Santa Cruz	Cat# sc-40
ANTI- RNA pol II CTD phospho Ser5	Active Motif	Cat#61086
ECL anti-mouse IgG from sheep	Life Technologies	Cat#NA931
ECL anti-Rabbit igG from goat	Life Technologies	Cat#A10518
Chemicals, peptides, and recombinant proteins		
Hydroxyurea	Sigma-Aldrich	Cat#H8627
Camptothecin	Sigma-Aldrich	Cat#C9911
Methyl methane sulfonate	Sigma-Aldrich	Cat#129925-5G
Proteinase K	Sigma-Aldrich	Cat#P6556
Nuclease S7, micrococcal nuclease from S. aureus	Sigma-Aldrich	Cat#10107921001
Glycine	Sigma-Aldrich	Cat#G8898
Formaldehyde	Sigma-Aldrich	Cat#F8775
Sodium Azide	Sigma-Aldrich	Cat#71289
Trioxsalen	Sigma-Aldrich	Cat# T6137
Phenol:Chloroform:Isoamyl Alcohol 25:24:1	Sigma-Aldrich	Cat#P2069
cOmplete mini-tablet	Roche	Cat#5056489001
a-factor	GenScript	Cat#59401-28-4
RNase A DNase-free	QIAGEN	Cat#79254
TB Green Premix Ex Taq II (Tli RNase H Plus)	TAKARA	Cat# RR82WR

Zymolyase 20T	MP Biomedicals	Cat#08320921
Biotin Azide (PEG4 carboxamide-6-Azidohexanyl Biotin)	Life Technologies	Cat# B10184
Alexa Fluor® 647 azide, triethylammonium salt	Life Technologies	Cat#A10277
Paraformaldehyde	Life Technologies	Cat# 047317.9L
Sytox Green	Life Technologies	Cat#S7020
Dynabeads protein G	Life Technologies	Cat#100.04D
Propidium Iodide	Life Technologies	Cat#P3566
5-Ethynyl-2’-Deoxyuridine (5-EdU)	Jena Biosciences	Cat#CLK-N001-100
Kits		
Genomic-tip 100/G	QIAGEN	Cat#10223
MSB® Spin PCRapace	INVITEK Molecular	Cat#1020220300

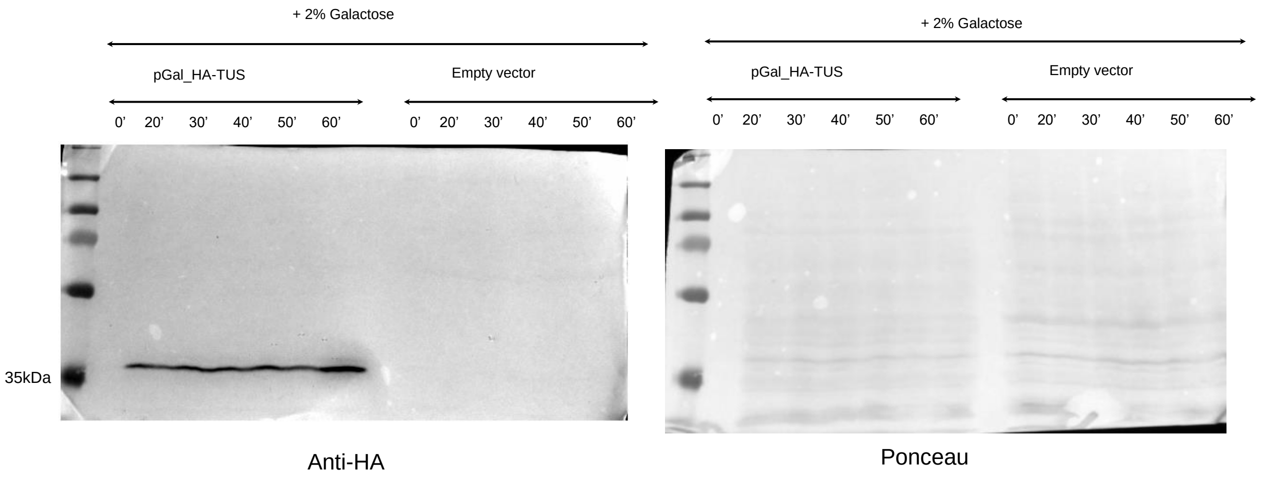

Western Blot showing Tus expression (uncropped)

Uncropped Western blot, related to Supp. Fig. 1
